# Therapeutic benefit of *Salmonella* attributed to LPS and TNF-α is exhaustible and dictated by tumor susceptibility

**DOI:** 10.18632/oncotarget.16906

**Published:** 2017-04-06

**Authors:** Dino Kocijancic, Sara Leschner, Sebastian Felgner, Ronja-Melinda Komoll, Michael Frahm, Vinay Pawar, Siegfried Weiss

**Affiliations:** ^1^ Department of Molecular Immunology, Helmholtz Centre for Infection Research, Braunschweig, Germany; ^2^ Institute of Immunology, Medical School Hannover, Hannover, Germany

**Keywords:** TNF alpha, LPS, cancer immune therapy, salmonella, bacteria mediated tumor therapy

## Abstract

The potential of bacteria-mediated tumor therapy (BMTT) is highlighted by more than a century of investigation. Attenuated *Salmonella* has prevailed as promising therapeutic agents. For BMTT - categorized as an immune therapy - the exact contribution of particular immune reactions to the therapeutic effect remains ambiguous. In addition, one could argue for or against the requirement of bacterial viability and tumor targeting. Herein we evaluate the isolated therapeutic efficacy of purified LPS and TNF-α, which together account for a dominant immunogenic pathway of gram negative bacteria like *Salmonella*. We show that therapeutic efficacy against CT26 tumors does not require bacterial viability. Analogous to viable *Salmonella* SL7207, tumor regression by a specific CD8^+^ T cell response can be induced by purified LPS or recombinant TNF-α (rTNF-α). Conversely, therapeutic effects against RenCa tumors were abrogated upon bacterial avitalization and limited using isolated adjuvants. This argues for an alternative mechanistic explanation for SL7207 against RenCa that depends on viability and persistence. Unable to boost bacterial therapies by co-injection of rTNF-α suggested therapeutic effects along this axis are exhausted by the intrinsic adjuvanticity of bacteria alone. However, the importance of TNF-α for BMTT was highlighted by its support of tumor invasion and colonization in concert with lower infective doses of *Salmonella*. In consideration, bacterial therapeutic effectiveness along the axis of LPS and TNF-α appears limited, and does not offer the necessary plasticity for different tumors. This emphasizes a need for recombinant strengthening and vehicular exploitation to accommodate potency, plasticity and distinctiveness in BMTT.

## INTRODUCTION

Tumor therapy using bacteria represents a concept with a long standing tradition [1]. Infection with various bacterial agents, alone or in combination, became an early strategy to counteract tumor growth [2, 3]. Pioneers such as William B. Coley deployed strategies of local application and/ or heat killing of bacteria to control infection while hoping to retain the therapeutic effect. Concurrently he made an early observation of a correlation between fever induction and a positive response [4].

Nowadays, explanations for the observed phenomena are partially at hand. Pathogenicity of bacteria and their dissemination accounted for the severe side effects through activation of acute innate immune responses, including a pyrogenic effect. In accordance, the field explored and promoted bacteria with intrinsic safety [5]. The gram-negative facultative anaerobic bacterium *Salmonella enterica* serovar Typhimurium (*S*. Typhimurium) prevailed as a most exploited bacterium for bacteria mediated tumor therapy (BMTT), exhibiting intrinsic therapeutic efficacy and specificity of tumor colonization [1, 6–9]. However, its application necessitates further attenuation.

Numerous reports have validated the tumor targeting ability of attenuated *Salmonella* across a wide range of different tumor models [10–15]. Our understanding grants *Salmonella* an ability to invade solid tumors via a passive mechanism. A chronic infection is established of above 1 × 10^8^ CFU with a specificity for tumors of above 1000:1 compared to normal murine tissue [16, 17]. In addition, the vehicular potential of *Salmonella* has been exploited to specifically deliver therapeutic cargo to a great variety of tumors with an astonishing degree of success [5, 18–22]. Meanwhile, the requirement of tumor-specific colonization for an intrinsic therapeutic effect remains ambiguous.

Pathogenesis of virulent gram-negative bacteria is induced via microbial associated molecular patterns (MAMPs) activating signaling cascades via surface-bound pattern recognition receptors (PRRs). The most prominent of these is Toll like receptor 4 (TLR4) recognizing Lipopolysaccharide (LPS) located on the outer membrane of gram-negative bacteria [23]. In response to systemic application of LPS, a cytokine storm is elicited that leads to an extensive physiological reaction including the activation and mobilization of immune cells that may aid and embed a tumor therapeutic effect [24–26].

Tumor necrosis factor -alpha (TNF-α) is a main cytokine induced along the LPS/ TLR4 axis, and is essential for the induction of septic shock [27]. Predominantly released by innate immune cells including macrophages, the effects of TNF-α are pleiotropic [28]. These include a pyrogenic effect, thus creating a hypothetical link to the observations of William B. Coley that fever is induced upon bacterial application, and most likely required for effective BMTT in humans.

A first described effect of TNF-α is the induction of necrosis in tumors, thus explaining its denotation [27, 29]. This property has rendered TNF-α a reliable read-out in various preclinical tumor models as well as a potential therapeutic adjuvant [30–32]. However, its stand-alone therapeutic capacity and level of contribution in BMTT remain intriguing questions, particularly considering the detrimental effects of this pro-inflammatory cytokine.

It has long been speculated that bacterial immunogenicity or adjuvanticity accounts for the intrinsic therapeutic effect of *Salmonella*, albeit alternate mechanisms involving tumor colonization or direct toxicity may also contribute. In support, bacterial intrinsic therapeutic effects were shown to depend on TLR4 and myD88, thus arguing for an immune based therapeutic mechanism [33, 34]. Later studies further demonstrated that bacteria stimulate an anti-tumor immune response and induce immunological memory. In particular, CD8^+^ cytotoxic T cells are predominantly involved in such therapeutic effects although CD4^+^ T cells may also contribute [35, 36]. It has been suggested that the adjuvant effect of *Salmonella* can be ascribed to LPS (or other MAMPs) alone and/ or its induction of TNF-α. This greatly underpins the scrutiny to BMTT. Addressing these questions is of importance, particularly considering the efforts made to tailor the intrinsic therapeutic effect of *Salmonella* via alteration of their LPS structure [37–39].

In the present work, we reevaluate SL7207, an attenuated variant of *Salmonella* Typhimurium, of which the therapeutic properties and mechanism have been extensively characterized [16, 17, 35, 40]. Using an approach of minimizing therapy, we asked the question to which extent therapeutic effects can be induced by purified LPS or recombinant TNF-α relative to viable SL7207. Our evaluation takes use of the colon and renal carcinoma models CT26 and RenCa, respectively. These were shown to exhibit differential susceptibility to *Salmonella*-mediated tumor therapy [36], thus providing a framework to assess the therapeutic plasticity of minimized therapies.

## RESULTS

### Tumor regression is inducible by LPS

Live bacteria underlie the majority of preclinical investigations concerning BMTT and, more recently, even clinical trials [41, 42]. It may have generated the impression that viable bacteria are a prerequisite for efficacy in this type of therapy. In our own work, this includes investigations using viable *S*. Typhimurium SL7207 and the transplantable colon carcinoma CT26. Thus, an important question remained, namely: to which extent does LPS or the downstream effector molecule TNF-α constitute components responsible for an intrinsic bacterial anti-tumor effect?

**Figure 1:**
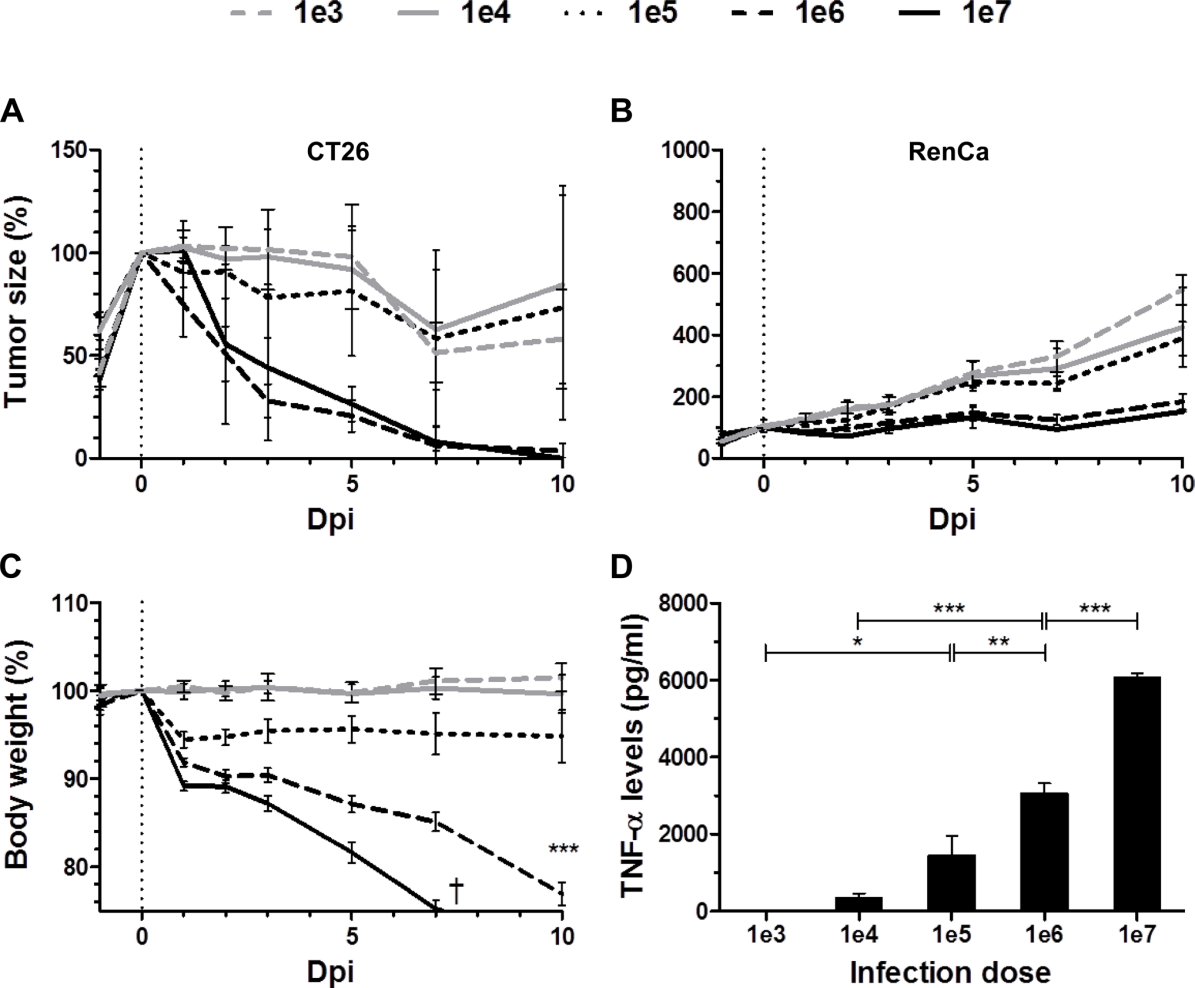
Dose-response relationship between SL7207 and therapeutic effects Tumor-bearing mice were inoculated i.v. with titrated doses of viable SL7207, as indicated. (**A**) CT26 tumor development profiles. *N* = 5. (**B**) RenCa tumor development profiles *N* = 6. Therapeutic potency increases with higher infection dose. (**C**) Host body weight profiles. *N* = 5. Increase in body weight loss with higher infection dose. (**D**) Levels of TNF-α in serum 1.5 hpi, as determined using ELISA. *N* = 5. Levels of TNF-α increase with higher infection dose. Displayed are Means ± SEM. Asterisks *, ** and *** denote significance levels of *p* < 0.05, *p* < 0.01, or *p* < 0.001, resp.

In a first step we determined if SL7207 mediated therapeutic effects are titratable. If so, it could imply a direct correlation with immunogenic components like bacterial PAMPs. As expected, development of both CT26 and RenCa tumors was influenced by infection. However the therapeutic potency was dependent on the dose of infection (Figure 1A, 1B). Only an infection dose of above 1 × 10^6^ caused persistent CT26 tumor regression in all replicates (15/15 with 1 × 10^6^ vs 9/15 with 1 × 10^5^), and a strong retardation of RenCa growth (2-fold decrease with 1 × 10^6^ and 1 × 10^7^ compared to 1 × 10^5^ at 10 dpi). As expected, the health status of the host worsened with higher infection dose of *Salmonella*. Morbidity and fatal loss of body weight were consequences of an infection dose of 1 × 10^7^ (Figure 1C), thus establishing a dose limitation in our model with SL7207.

As a measure of innate immune reactions, we measured the systemic TNF-α response with altering doses of salmonellae. The serum levels of TNF-α directly correlated with the infection dose of SL7207 (Figure 1D). As TNF-α is a known downstream mediator of bacterial PAMPs, particularly of Lipopolysaccharide (LPS), we hypothesized that LPS per se would induce similar therapeutic effects.

**Figure 2:**
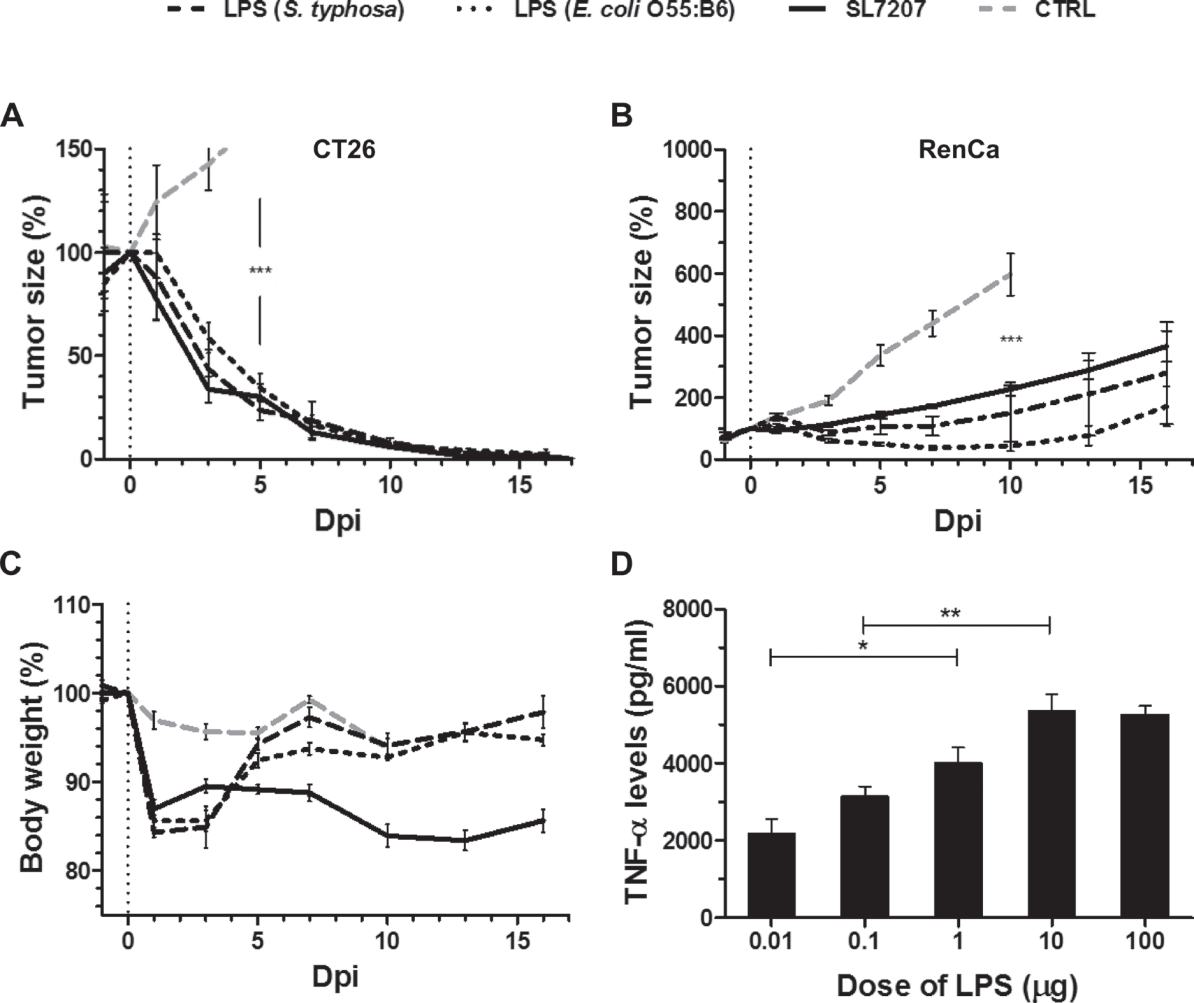
Single-dose purified LPS induces CT26 clearance and exhibits transient potency against RenCa Tumor-bearing mice were inoculated i.v. with 5 × 10^6^ viable SL7207 or 50 μg purified LPS from *Salmonella typhosa or E. coli* O55:B6, before comparing the therapeutic outcomes. (**A**) CT26 tumor development profiles. *N* = 5. Treatments display a comparable kinetic of tumor regression. (**B**) RenCa tumor development profiles *N* = 6. Transient therapeutic benefit of systemically inoculated LPS. (**C**) Host body weight profiles. *N* = 5. Transient weight loss in response to purified LPS, distinct from progressive weight loss during salmonellosis. (**D**) Levels of TNF-α in sera at 1.5 hpi, as determined using ELISA. *N* = 3. Dose-response to LPS. Displayed are Means ± SEM. Asterisks *, ** and *** denote significance levels of *p* < 0.05, *p* < 0.01, or *p* < 0.001, resp.

To determine the isolated contribution of LPS, we deployed single-dose systemic injection of purified LPS to tumor-bearing mice and evaluated the effects on CT26. Surprisingly, CT26 tumor development was completely abrogated. The kinetic of regression was similar to viable SL7207 (Figure 2A). To generalize effects, LPS molecules from different bacterial sources were deployed (i.e. *S*. *typhosa* and *E. coli* O55:B5), all of which yielded comparable results (Figure 2).

In contrast, with more resistant RenCa tumors purified LPS induced only transient growth regression using sub-lethal doses (Figure 2B, Supplementary Figure 1). This would suggest that LPS alone is inadequate to clear this type of tumor. Of note, also SL7207 caused at best only retardation in the RenCa system, highlighting that this particular type of tumor exhibits greater general resilience to BMTT.

The mere transient effect of purified LPS was reflected in the body weight profile of the murine hosts. A weight loss of 15% on day 1–3 post inoculation led to normalization by 5 dpi (Figure 2C). In line with current dogma explaining an endotoxic effect, application of LPS induced a TNF-α response. The kinetic profile peaking at 1.5 hpi was similar to the systemic response to bacteria described before (Supplementary Figure 2) [16], and moreover titratable (Figure 2D). Altogether, the host body weight, tumor development, and the TNF-α response were affected similarly by titrated doses of *Salmonella* and purified LPS (Figures 1, 2, Supplementary Figure 1). Similarity between the therapeutic profiles would imply that bacterial therapeutic effects are indeed elicited along this axis, albeit more PAMPs and other mechanisms could contribute in the case of bacteria.

**Figure 3:**
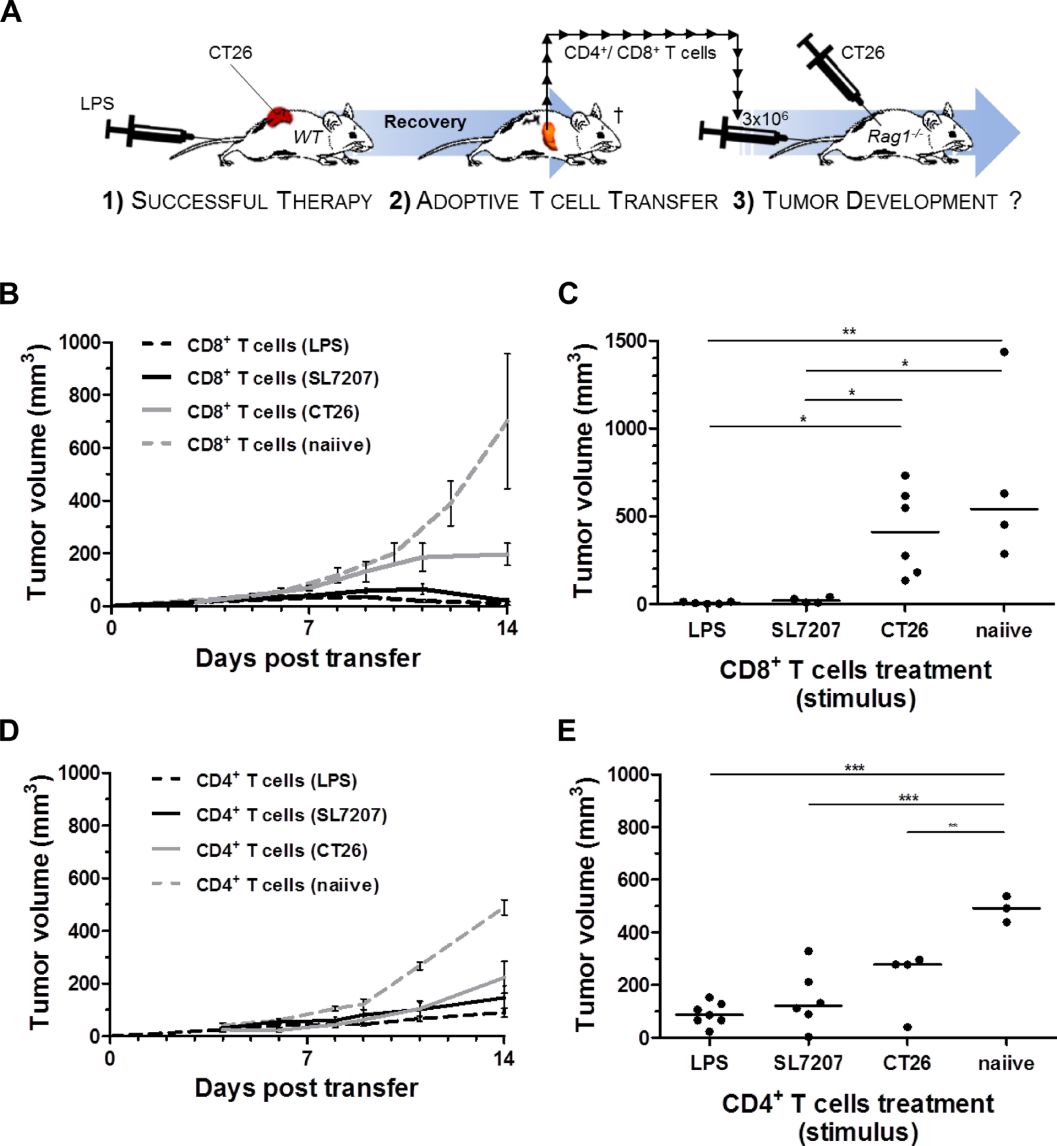
Analogous to SL7207, purified LPS elicits a tumor-specific CD8^+^ T cell response capable of CT26 rejection Therapeutic effects of pre-stimulated CD8^+^ or CD4^+^ T cell populations were evaluated on CT26 tumor growth in Rag1^−/−^ mice. (**A**) Schematic depiction of reconstitution experiments performed. In essence, 1) durable and complete regression of CT26 tumors through primary therapy (here purified “LPS”) in wild-type mice is a prerequisite for T cell isolation. 2) Upon recovery; 3 months post therapy and antibiotic (ciprofloxacin) treatment [77], 3 × 10^6^ CD4^+^ or CD8^+^ T cells are adoptively transferred to Rag1^−/−^mice following negative purification from a pool of splenocytes recovered from the wild-type donor. 3) Concurrent s.c. injection of 5×10^5^ CT26 tumor cells allows for evaluation of tumor growth and -establishment post adoptive transfer. (**B**) CT26 tumor development in Rag1^−/−^mice reconstituted with CD8^+^ T cells from wild-type donors pre-stimulated as indicated in the brackets. *N* = 6. (**C**) End-point significance comparison of mean tumor volumes in Rag1^−/−^mice upon reconstitution with pre-stimulated CD8^+^ T cells. Reconstitution with LPS stimulated CD8^+^ T cells prevents establishment of CT26 tumors (**D**) CT26 tumor development in Rag1^−/−^mice reconstituted with CD4^+^ T cells from wild-type donors pre-stimulated as indicated in the brackets. *N* = 6. (**E**) End-point significance comparison of mean tumor volumes in Rag1^−/−^mice upon reconstitution with pre-stimulated CD4^+^ T cells. No significant difference to the CT26 control. The (CT26)-group denotes presence of an established, albeit untreated, CT26 tumor on the T cell donor. Displayed are Means ± SEM. Asterisks *, ** and *** denote significance levels of *p* < 0.05, *p* < 0.01, or *p* < 0.001, resp.

We have previously shown that SL7207 stimulates a tumor specific CD8^+^ T cell response capable of rejecting CT26 tumors [35]. Mice that recovered from CT26 upon single LPS treatment were analogously resistant to re-challenge with CT26 (Supplementary Figure 3), indicating that a tumor specific memory response had been evoked. We next asked if CD8^+^ T cells are activated via LPS treatment. To address this question, we performed a series of adoptive transfer experiments in Rag1^−/−^ mice using an established protocol (Figure 3A) [35, 36]. Purified LPS induced a pool of CD8^+^ T cells capable of preventing CT26 tumor establishment in Rag1^−/−^mice (Figure 3B). By 14 days post reconstitution with CD8+ T cells, individual tumor masses were significantly reduced below measurable and palpable sizes (Figure 3C). As seen before, the mere presence of a CT26 tumor in a mouse already stimulated a tumor specific CD8^+^ T cell response capable of retarding tumor growth in reconstituted Rag1^−/−^mice (Figure 3B; group: “CD8^+^ T cells (CT26)”). However, net tumor volumes 14 days post transfer revealed that a proportion of these recipients were unable to control the outgrowth of the tumor (Figure 3C). This suggests that the pre-stimulation by CT26 alone might not result in a sufficiently high number of tumor-reactive CD8^+^ T cells required to counteract the tumor in every case. Similar reconstitution experiments using CD4^+^ T cells did result in limited growth retardation under similar conditions. However, equal efficiency between different groups (“LPS”, “SL7207” and “CT26”) argued for an insignificant influence on CD4^+^ T cells by purified LPS alone. (Figure 3D, 3E).

Taken together, systemic LPS therapy alone is sufficient to enhance a tumor-specific CD8^+^ T cell response as required for CT26 tumor rejection. This highlights a mechanistic explanation for the therapeutic activity of gram-negative bacteria like *Salmonella* SL7207. However, the strength of the response elicited by LPS alone only suffices to impede the growth of resistant tumors like RenCa while it remains insufficient to clear them.

### A TNF-α response underlies effective treatment of CT26

**Figure 4:**
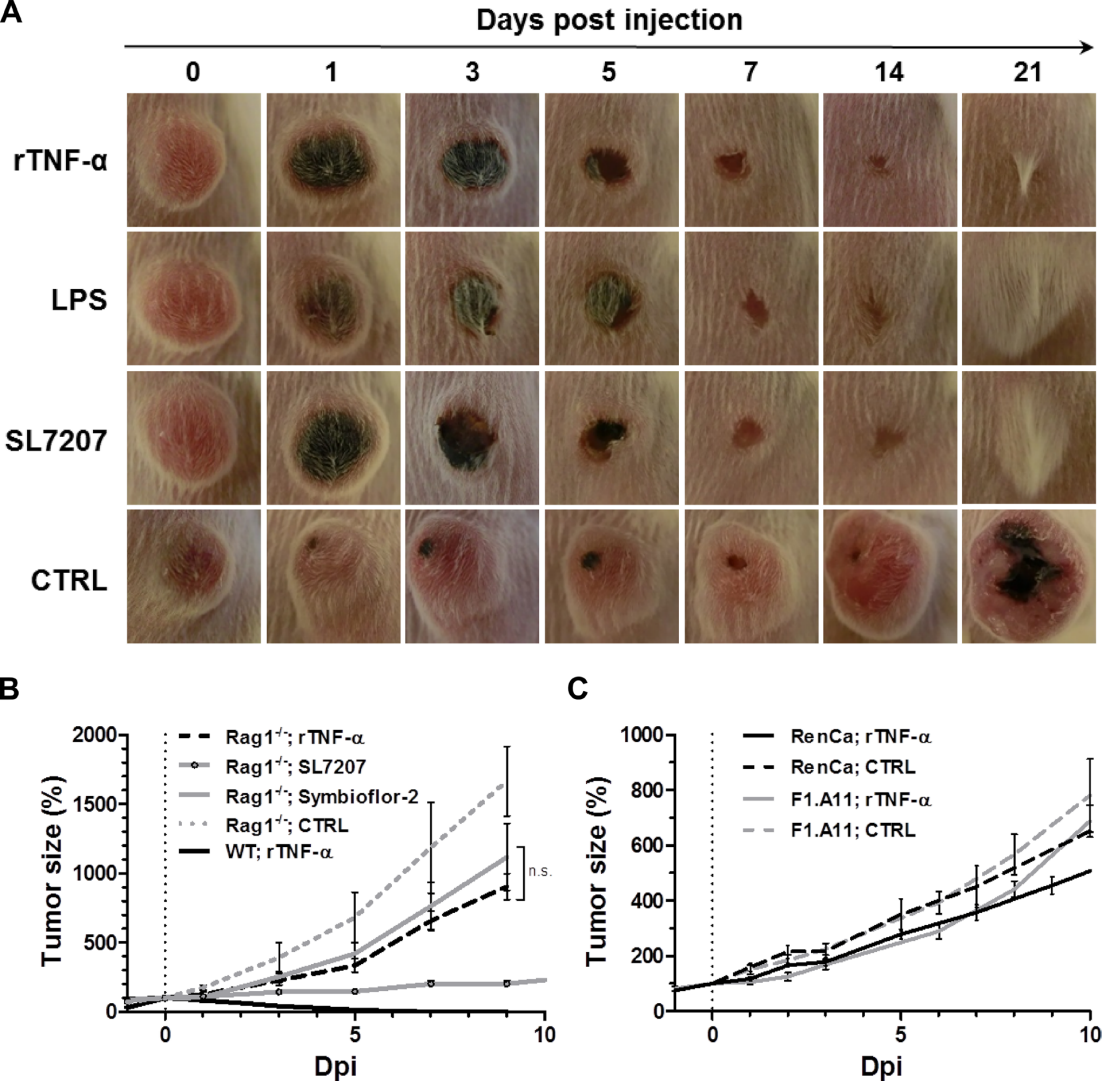
While exclusive to CT26, tumor clearance is equally attainable through recombinant TNF-α as with LPS or *Salmonella*, and dependent on adaptive immunity 1 μg recombinant murine TNF-α (rTNF-α) was administered i.v. and therapeutic effects in wild-type and Rag^−/−^mice were evaluated relative to LPS therapy or BMTT. (**A**) CT26 tumor development in WT mice represented by close-up photographs of CT26 tumors subjected to treatments as indicated. Similar macroscopic profile of tumor darkening, ulceration and clearance between all therapies. Images displayed are representative of three individual replicates (**B**) Kinetic of CT26 tumor development in Rag1^−/−^mice. *N* = 5. Tumor regression with all therapies is abrogated in absence of a functional adaptive immune system. (**C**) RenCa and F1.A11 tumor development profiles in WT mice. *N* = 5. No significant therapeutic effects induced by rTNF-α. Displayed are Means ± SEM.

Considering the reported role of TNF-α and its induction via the LPS/ TLR4 axis, we asked whether this central cytokine alone may induce effects similar to LPS in the CT26 tumor model. Indeed, systemic treatment using recombinant TNF-α (rTNF-α) caused CT26 tumor regression in wild-type mice with a macroscopic developmental profile similar to previously tested more complex therapies like bacteria (SL7207) or the upstream stimulant LPS (Figure 4A). 1 dpi, darkening of the tumor tissue was evident, resulting in ulcerative necrosis over the following 3–5 days. This manifestation is symptomatic for the originally described effect of TNF-α and fitting with previous reports for SL7207 [16, 40]. Here, this phenotype was followed by complete tumor regression and wound healing by 3 weeks after injection.

Abrogation of the therapeutic effect in lymphopenic Rag1^−/−^ mice, suggested that the therapeutic effect of rTNF-α depends on a functional adaptive immune system (Figure 4B) [43, 44]. This may likely be explained by an analogous adjuvant effect on CD8^+^ T cells as demonstrated earlier for LPS (Figure 3). In support of a memory response, also mice having cleared CT26 tumors through recombinant TNF-α were resistant to re-challenge with CT26 (Supplementary Figure 3). In Rag1^−/−^mice, rTNF-α displayed only a tendency of therapeutic effects at a level similar to probiotic *E. coli*, and far from the significant growth retardation observed with the more potent strain SL7207 (4 fold decrease in tumor size compared to rTNF-α or Symbioflor-2 at 9 dpi) (Figure 4B). Thus, B/T cell independent effects are only stimulated to a minor degree by rTNF-α, and inferior to the effects along an anti-tumor adaptive immune response. As single-agent therapy in wild-type mice rTNF-α induced a substantial therapeutic effect only on CT26, and failed to yield significant therapeutic benefit with both RenCa and another highly resistant fibro-sarcoma F1.A11 (Figure 4C).

### Bacteria and inflammatory mediators share characteristics of solid tumor therapy

**Figure 5:**
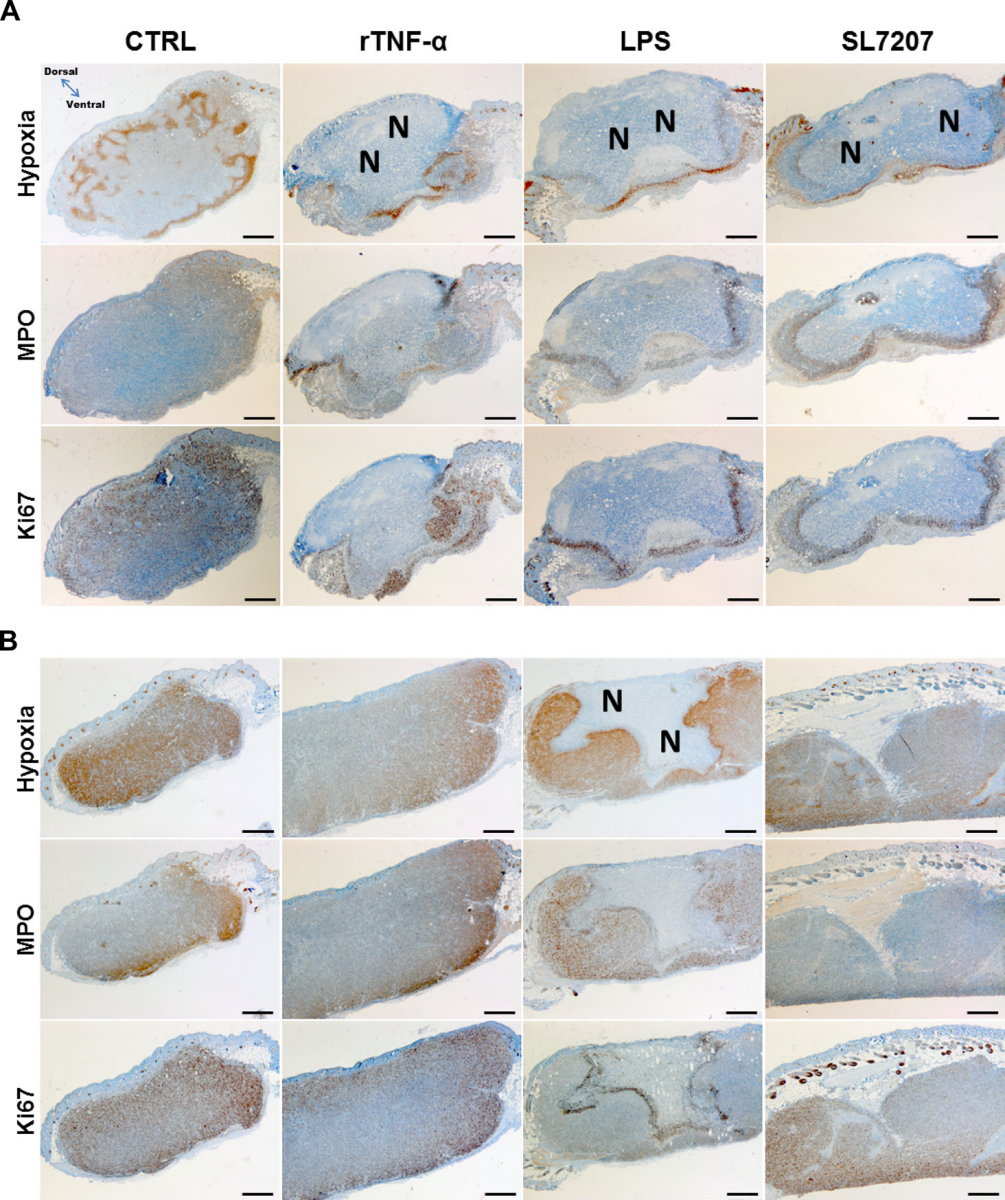
Prognostic characteristics of the CT26 tumor are shared among different immunogenic treatments, and less pronounced in RenCa Tumors from treated or untreated mice were isolated 48 hpi, and subjected to immune histochemical staining. Histological images of stained CT26 (**A**) or RenCa (**B**) tumor cross sections. More extensive necrosis formation with treatments of CT26 compared to RenCa. Images displayed are representative of four replicates. “N” indicates greater areas of necrosis. Hypoxia was stained with antibodies against metabolites of pimonidazole-HCl, otherwise administered i.v. 30 mins prior to isolation. Myeloperoxidase (MPO) denotes presence of neutrophilic granulocytes, and Ki67 the extent of proliferative activity. Differential staining was performed on consecutive sections. Scale bar corresponds to 100 μm. Orientation of the tumor cross section as indicated.

To cast more light on the early therapeutic effects and differences in therapeutic outcomes among tumor models and treatments, we analyzed the microscopic profile of differentially treated tumors. Interestingly, CT26 tumor cross sections isolated 48 h post treatment with rTNF-α or LPS bore similar phenotypic manifestations. Control tumors were characterized by more widespread areas of hypoxia and greater dispersion of residing neutrophils and proliferative activity. All immunogenic treatments caused extensive necrosis formation towards the dorsal side of the tumor, thereby confining and localizing the neutrophils to the viable border at the remaining basal part (Figure 5A). This phenotype correlated with the macroscopic profile of tumors displayed earlier (Figure 4A). Considering the consistent manifestation of necrosis with all effective treatments against CT26, one may substantiate such as a prognostic factor in this tumor model.

Hypothesizing the same may hold true in RenCa, we repeated our microscopic analysis with this model. RenCa generally contained more areas of hypoxia, a greater extent of proliferative activity, and less pronounced necrosis formation compared to that encountered in CT26 (Figure 5B). However, with LPS, necrosis was not as deteriorating as observed in CT26, leaving a larger viable part unaffected. This by itself could explain a re-establishment of the tumor. Neither recombinant TNF-α nor SL7207 displayed a marked difference compared to the control. Thus no additional clues are provided as to how SL7207 exerts its therapeutic (i.e. growth retarding) effect.

To identify the origin of the increased and localized proliferative activity, higher magnification images of the IHC staining allowed evaluating the cell morphology. While a smaller proportion of the Ki67 positive cells may constitute innate effector cells, the vast majority appeared to originate from hypertrophic tumor cells most adjacent to the necrosis (Supplementary Figure 4).

### Viability of *Salmonella* as differential requirement for successful therapy of CT26 and RenCa tumors

**Figure 6:**
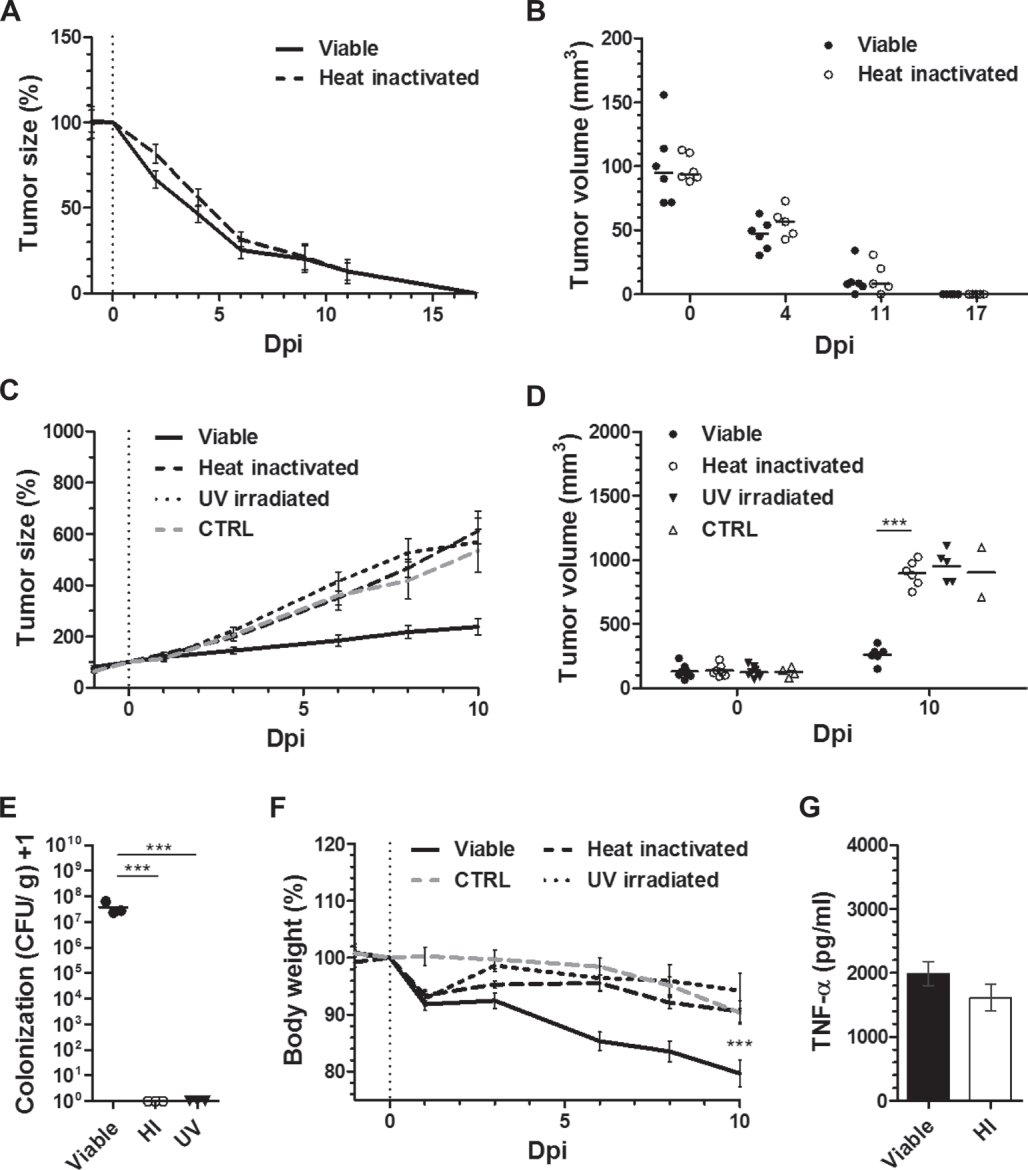
While dispensable for CT26 regression, viability of SL7207 is cause of an infectious phenotype, and required for the therapeutic efficacy against RenCa Tumor-bearing mice were inoculated i.v. with 5 × 10^6^ viable or avitalized *Salmonella* SL7207, and the therapeutic effects compared. Avitalization was facilitated through heat inactivation (HI) at 60°C or UV irradiation. (**A**) CT26 tumor development profiles. *N* = 6. Mean ± SEM. Similar kinetic of regression post treatment using viable and avitalized SL7207. (**B**) Statistical comparison between mean tumor volumes. No difference at indicated time points. (**C**) RenCa tumor development profiles. *N* = 6. Mean ± SEM. In contrast to viable SL7207, avitalization abrogates the retarding effect and causes outgrowth. (**D**) End-point comparison of mean RenCa tumor volumes. No significant difference between UV irradiated and HI groups. (**E**) CFU counts in RenCa tumors upon 48 hours of treatment, as indicated. *N* = 3. Median ± range. Only viable therapy facilitates tumor colonization (i.e. persistence) of SL7207 in the host. (**F**) Host body weight profile dictated by recovery for avitalized groups, and progressive loss with viable SL7207. *N* = 6. Mean ± SEM. (**G**) Levels of TNF-α in sera at 1.5 hpi, as determined using ELISA. *N* = 5. Mean ± SEM. No significant difference between viable and avitalized treatments. Asterisks *, ** and *** denote significance levels of *p* < 0.05, *p* < 0.01, or *p* < 0.001, resp.

The minimization of stimulants for tumor therapy proved only successful for CT26 which is evidently prone to this type of immune therapy. We therefore increased the complexity of the stimulant and investigated the therapeutic effect of heat-inactivated *S*. Typhimurium SL7207 on tumor development. Heat-inactivated *Salmonella* would still bear most of the stimulating molecules [45]. As expected, avitalized SL7207 induced CT26 tumor clearance with a kinetic of regression similar to viable SL7207 (Figure 6A). Endpoint tumor volumes were indifferent among the groups, reaching mean tumor sizes of < 10 mm^3^ by 11 dpi and non-palpable tumors by 17 dpi (Figure 6B). These results were corroborated with an independent bacterial agent, the *E. coli* probiotic Symbioflor-2, also displaying comparable tumor retardation profiles between viable and avitalized bacterial treatment (Supplementary Figure 5). Inability to re-establish CT26 tumors upon re-challenge after successful therapy with HI SL7207 (Supplementary Figure 3), supports that immune memory is likely to be elicited along the same axis as viable bacteria, purified LPS or rTNF-α. The positive therapeutic outcome on CT26 tumors by avitalized bacteria again correlated with the histological manifestation of necrosis, analogous to treatments with both viable bacteria and LPS (Supplementary Figure 6).

To validate our results and determine if viability may just as easily be surpassed in other tumor models, we tested RenCa. In addition to heat-inactivation we included avitalization by UV irradiation. Surprisingly, and regardless the mode of avitalization, both heat-inactivation and UV irradiation completely abrogated the therapeutic activity of SL7207 against RenCa tumors (Figure 6C). Endpoint tumor volumes showed no significant difference between treatments with avitalized SL7207 and PBS (Figure 6D). As expected, no presence of avitalized bacteria could be detected in the tumor upon control plating (Figure 6E), thus dissociating the bacterial therapeutic effects observed on CT26 from bacterial persistence or presence in the tumor. However, lack thereof may still contain possible explanations for inefficiency against RenCa.

Evaluating the body weight over the course of treatment, avitalization altogether abrogated the detrimental effect of viable bacteria on the host. Compared to the therapy with SL7207, which induced a progressive drop in body weight by 11 dpi, mice treated with heat-inactivated or UV irradiated SL7207 retained an inclining body weight profile after a transient drop of < 10% by 2 dpi (Figure 6F). This result correlated with the phenotypic appearance of the animals. In line with earlier reports [36], mice subjected to viable infection displayed fatigue and a disrupted fur coat. This was in great contrast to those subjects inoculated with heat-inactivated SL7207 and not displaying any signs of behavioral or phenotypic change (Supplementary Figure 7).

Expecting that a systemic immune reaction might be similarly induced by heat-inactivated SL7207 and may underlie the therapeutic effect on CT26, we determined the amount of TNF-α in serum shortly after inoculation. A systemic TNF-α response was indeed detected early post inoculation (1.5 hpi). No significant difference to infection by viable bacteria was observed (Figure 6G). This indicates that also heat-inactivated SL7207 induces a substantial cytokine response that is already sufficient for CT26 clearance.

### Limited efficiency of recombinant TNF-α in solid tumor therapy

**Figure 7:**
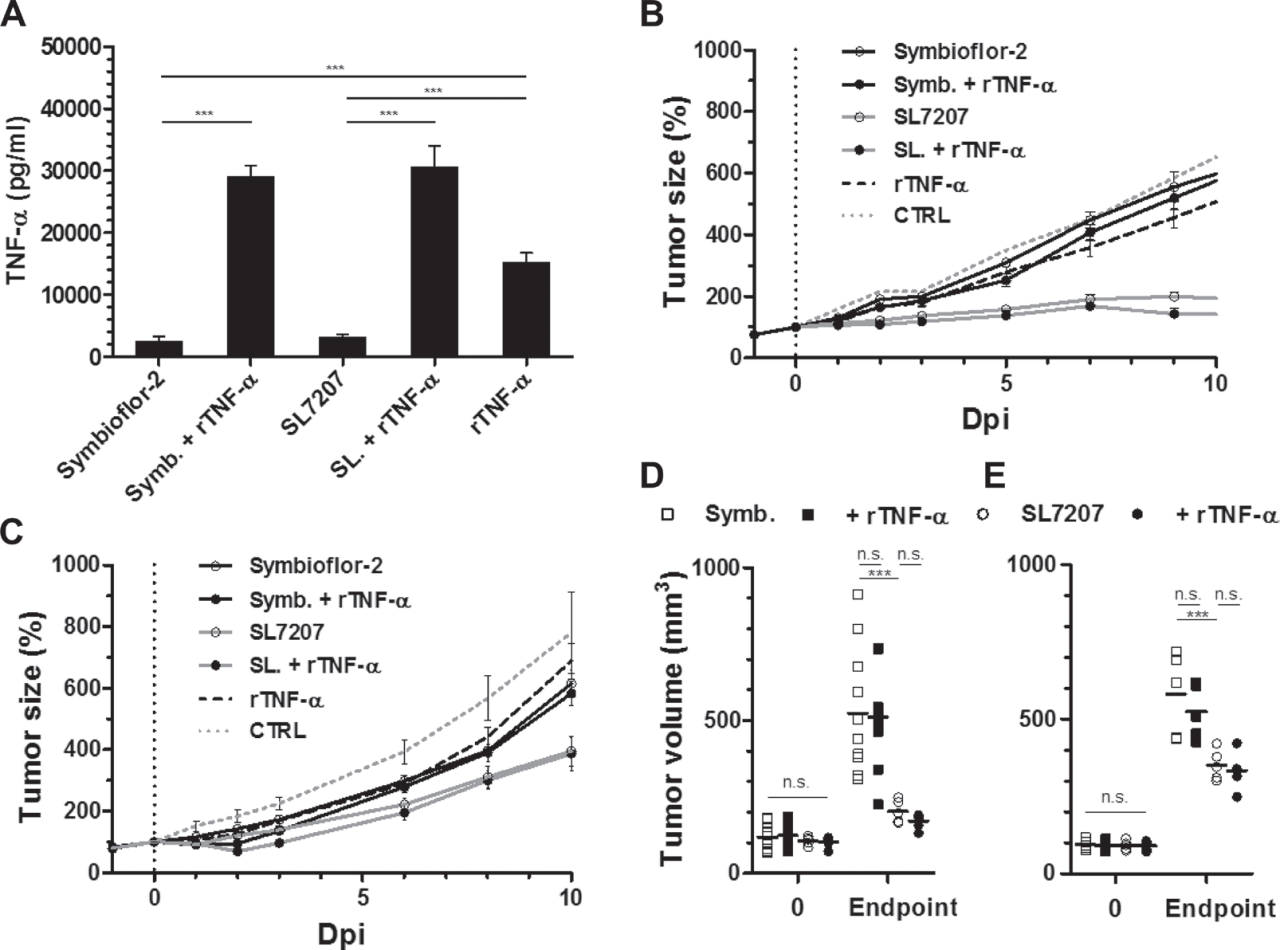
Co-injection of recombinant TNF-α does not improve bacterial anti-tumor effects in more rigid tumor models Tumor-bearing mice were inoculated i.v. with 5 × 10^6^ *Salmonella* SL7207 or *E. coli* probiotic Symbioflor-2 in concert with 1 μg recombinant TNF-α. Therapeutic efficacies were compared. (**A**) Levels of TNF-α in sera at 1.5 hour post treatment, as determined using ELISA. *N* = 5. Combination therapy yields excessive levels of TNF-α. (**B**) RenCa tumor development profiles in treated WT mice. *N* = 8. (**C**) F1.A11 tumor development in treated WT mice. *N* = 5. (**D**) Endpoint RenCa tumor volume. Comparison at 9 dpi. (**E**) Endpoint F1.A11 tumor volume. Comparison at 10 dpi. TNF-α supplementation does not improve bacterial therapeutic effects. Displayed are Means ± SEM. Asterisks *, ** and *** denote significance levels of *p* < 0.05, *p* < 0.01, or *p* < 0.001, resp.

Although limited, TNF-α exhibited a certain therapeutic potential. We wondered if i) increasing TNF-α could be a mean to boost a therapeutic effect, and if ii) a synergistic effect would be elicited when bacteria and TNF-α are administered in combination. Utilizing the known differential therapeutic efficiencies of SL7207 and probiotic *E. coli* (Symbioflor-2) [36], we asked if recombinant TNF-α may boost BMTT in more rigid tumor models. Co-administration facilitated a substantial increase in the systemic level of TNF-α at 1.5 hpi, past the response to bacteria or inoculum of rTNF-α alone, suggestive of positive feedback (Figure 7A). However, combination therapy of rTNF-α and Symbioflor-2 did not influence the RenCa tumor development relative to Symbioflor-2 alone, and also the tumor retarding effect of SL7207 did not improve in combination with rTNF-α (Figure 7B). These results were corroborated using a different tumor model F1.A11 with a similar outcome (Figure 7C). Endpoint tumor volumes did not display significant differences in either model upon co-injection of TNF-α (Figure 7D, 7E). Moreover, these results were transferrable to Rag1^−/−^ mice (data not shown).

Taken together, these results suggest that effector mechanisms induced by TNF-α at the therapeutic phase of BMTT may already be driven to its limit by the bacteria alone and that no additional effect can be achieved when recombinant TNF-α is applied in combination with the bacteria.

### Exogenous TNF-α supports tumor invasion under limiting conditions

**Figure 8:**
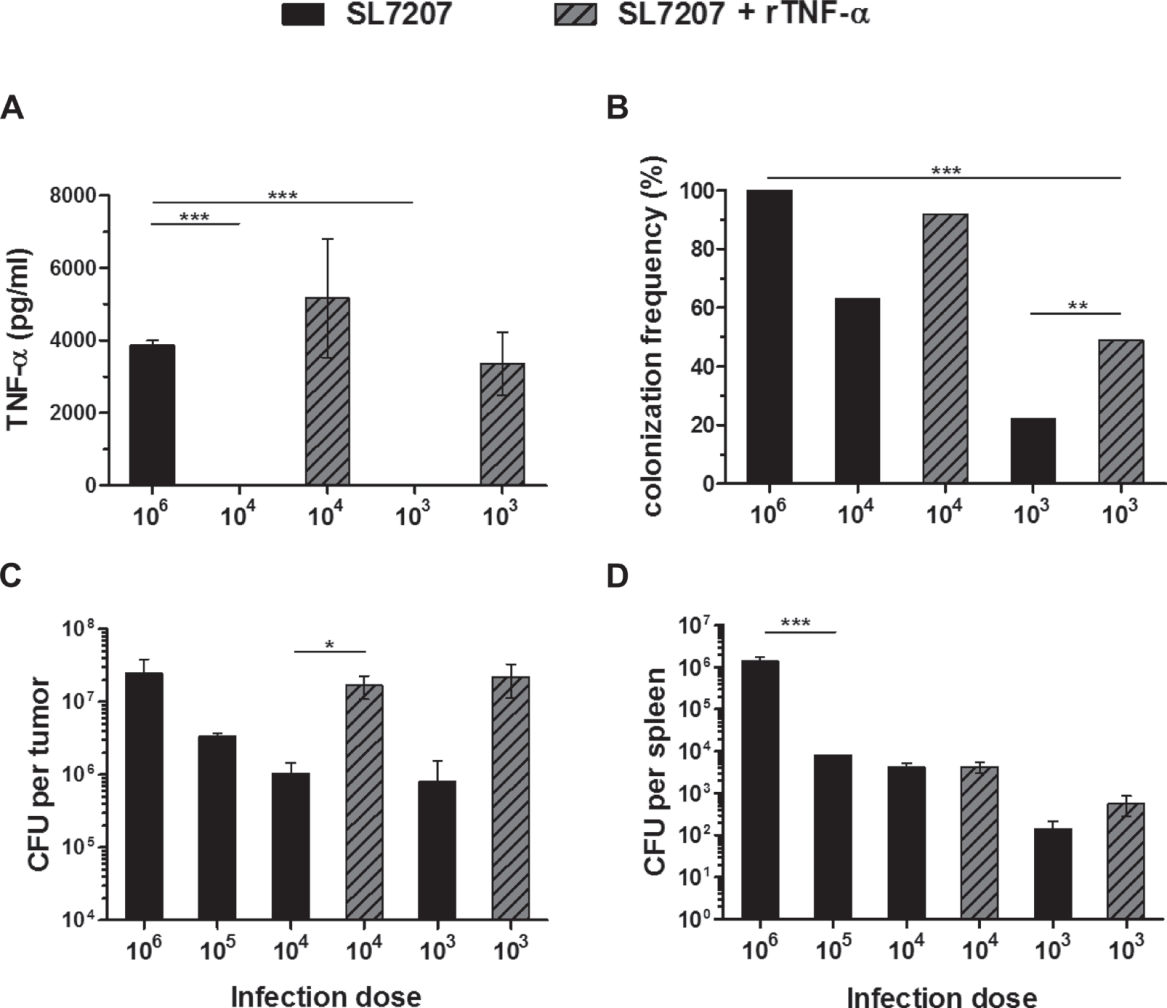
While the infectious dose of *Salmonella* decides about effectiveness of colonization, TNF-α supplementation supports tumor invasion at lower inoculi CT26 tumor-bearing mice were inoculated i.v. with *Salmonella* SL7207 alone or in combination with 1 μg recombinant TNF-α. Infectious doses were titrated from 5 ×10^6^, decreasing in increments of 1 log down to 5 ×10^3^. (**A**) Level of active TNF-α in sera as determined using a TNF-α sensitive C5F6 fibroblast bioassay. *N* = 5. Mean ± SEM. Lower doses fail to induce a significant TNF-α response. Exogenous rTNF-α can yield compensatory levels. (**B**) Frequency of colonized CT26 tumors. Results were pooled from three separate experiments. (**C**) CT26 tumor colonization 48 hpi. Tumors not colonized (refer to Figure 8B) were excluded from this calculation. Displayed are Medians ± range. Lower doses substantially reduce the frequency and efficiency of tumor colonization, compensable through co-injection of rTNF-α. (**D**) Splenic colonization. *N* = 5. Median ± range. Gradual decrease in adverse colonization with lower infectious doses and no marked change upon supply of rTNF-α. Asterisks *, ** and *** denote significance levels of *p* < 0.05, *p* < 0.01, or *p* < 0.001, resp.

As demonstrated in our experiments using recombinant TNF-α, this cytokine supports the clearance of solid tumors to a certain extent. During the phase of bacterial invasion into the tumor its vascular disrupting activities might be essential, and are most likely responsible for the early retardation of tumor growth [16, 31, 46]. In consideration, we tested the effects of exogenous TNF-α under conditions where the spontaneous induction of this cytokine is minimized. To this end, we reduced the number of bacteria in the inoculum. Supporting a dose-dependent relationship, serum levels of active TNF-α were reduced below detection limit early after infection with a 100-fold lesser inoculum of 5 × 10^4^ CFU (Figure 8A). However, an exogenous supply of rTNF-α installed comparable systemic levels, thereby hypothetically allowing for therapeutically beneficial activities compensatory to those of endogenously induced TNF-α. Using this setup, we determined the ability of *Salmonella* bacteria to invade CT26 tumors under minimized conditions. Compared to the standard inoculum of 5 × 10^6^, lower doses of 5 × 10^4^ and 5 × 10^3^ resulted in colonization of only 63% and 22% of all CT26 tumors, respectively (Figure 8B). Additionally, the level of colonization per tumor at 48 hpi was decreased in a dose-dependent manner, resulting in more than a 10-fold reduction in tumor CFU with an infection dose of 5 × 10^4^ (Figure 8C). Surprisingly, the addition of exogenous rTNF-α reinstalled the colonization efficiency and partially improved the colonization frequency (Figure 8B, 8C). Interestingly, while adverse colonization of spleen was reduced with lower infection doses no marked change was observed upon supply of rTNF-α (Figure 8D).

As exogenous rTNF-α was able to partially rescue the therapeutic activity of bacterial tumor invasion, the importance of this cytokine could be illustrated in that, it needs to be induced to a certain concentration for BMTT to become effective.

## DISCUSSION

This study aimed to determine if tumor-therapeutic effects against CT26 and RenCa described for SL7207 can be equally induced via avitalized bacteria, LPS or recombinant TNF-α, likely accounting for the dominant adjuvant pathway of gram-negative bacteria. Moreover, we intended to define limits of these adjuvant effects. We found that regression of CT26 tumors can be equally accomplished via single application therapy using recombinant TNF-α or purified LPS. It resulted in the induction of an effective anti-tumor CD8^+^ T cell response. In contrast, therapeutic effects against RenCa were abrogated upon avitalization of SL7207, only transiently present using high-dose purified LPS, and altogether absent using excessive amounts of rTNF-α. This demonstrates a requirement of viability and persistence of bacteria for therapeutic efficacy against RenCa tumors. Co-injection of rTNF-α with *E. coli* probiotic Symbioflor-2 or SL7207 did not improve bacterial therapeutic effects thereby suggesting that adjuvant effects are exhausted already by the bacteria alone under these conditions. However, the ability of rTNF-α to rescue the efficiency of tumor colonization with lower infection doses emphasizes the importance of this cytokine for tumor invasion.

Our study explored the often stated hypothesis that BMTT using gram-negative bacteria can be reduced to a mere adjuvant effect stimulating an anti-tumor immune response exclusively along the LPS/ TLR4 axis. While this mechanistic pathway was shown to apply in some cases, our study clearly demonstrates that sufficiency of such an intrinsic effect is tumor model dependent. In addition B/T cell independent effects in Rag1^−/−^ mice could be observed with therapy using viable SL7207.

The ease of CT26 clearance via single-shot therapy using LPS and rTNF-α, and thus stimulation of a preexisting CT26-specific CTL response, would suggest an easily over-turnable immune status for this type of tumor [47]. Thus, CT26 is rendered highly susceptible to this type of immunotherapy. Similar macroscopic profiles and an identical kinetic of regression of CT26 obtained with all types of therapeutic stimulants support this notion. Alternatively, more resistant models, like RenCa, may more appropriately reflect the clinical challenge and allow to asses bacterial therapeutic effects more accurately [10, 48–50].

The explanation of this differential susceptibility to adjuvant therapy between CT26 and RenCa remains uncertain. As innate immune responses (i.e. TNF-α) to SL7207 or *E. coli* remained comparable between CT26 and RenCa (Supplementary Figure 8), one would not speculate a difference in such between CT26 and RenCa. Hence, it may be the extent of resistance or escape mechanisms e.g. immune editing or check-point inhibition versus immune recognition and availability of tumor antigen. Other explanations may be given by the tumor architecture e.g. vascularization and accessibility for immune cells. Our histological analyses suggested necrosis formation may be a prognostic factor for clearance of CT26 tumors, supporting current dogma [40, 51]. It showed that indeed this feature is attainable by minimized therapy using LPS or rTNF-α. However considering also the requirement of an adaptive immune response, one may speculate that the extent of necrosis formation in the induction phase is crucial to reduce the tumor mass to a level where the capacity of the immune system is able to outbalance the remaining tumor growth. The more prudent manifestation of necrosis in RenCa would support this theory, although complete absence with SL7207 argues for a different explanation or mechanism in case of bacteria. Here, the outgrowth in spite of retardation would suggest that the capacity of the anti-tumor immune response is exceeded. While successful clearance of RenCa may surely require supplementation in an extrinsic form [1, 5, 52, 53], the localized constant stimulation by persistent *Salmonella* sets it apart from single agent immune therapies externally delivered to the tumor.

A differential viability requirement was shown for CT26 and RenCa. Our results demonstrate that bacterial viability or persistence, hence tumor colonization, is expendable for the adjuvant effect. This raises several questions. i) At which location does the stimulation of tumor reactive T cells take place if not in the colonized tumor, and ii) do reactions against bacterial antigens and/ or tumor colonization add substantial benefit to a viable bacterial therapy. The latter may explain the difference with RenCa. To which extent bacterial tissue destruction, liberation of neo-antigen, local stimulation of immune cells and a persistent stimulus are important or represent mechanisms that substantially contribute a therapeutic effect remains to be demonstrated.

The frequent attention on neutrophils in relation to bacterial immune therapy may have generated the impression of such as an important mediator of a therapeutic effect [54, 55]. Analogously, the neutrophil containment of bacteria was brought forth by our research group as an important mechanism of control, and potential therapeutic aid via modulated and polarized activities [40, 56–58]. However, the presence of a neutrophil barrier around the induced necrosis was equally attainable by rTNF-α or LPS. While this may disprove a requirement of bacterial colonization for immigration of innate immune cells to the tumor, the impact of bacteria on the functionality of these and other innate effector cells, including macrophages and NK cells [59], remains ambiguous.

In accordance with current dogma, LPS was shown to be a potent MAMP of gram-negative *Salmonella*, and TNF-α a downstream cytokine alone capable of inducing tumor necrosis and reinforcing a specific adaptive immune response. Not surprising, these stimulants are in focus as potential therapeutic adjuvants or as molecular targets for immune modulation in BMTT [30, 60–62]. However, one should keep in mind their naturally detrimental effect. Compromised safety may prohibit the option of boosting effects by higher dosing or re-stimulation, thus limiting application regimens. Along this line, sub-lethal doses of LPS at best caused only a transient therapeutic effect in our study, in spite of inducing excessive amounts of TNF-α. This suggests that such a mechanistic axis has a limited therapeutic effect. What may certainly challenge the feasibility of application is the consideration that humans, in downstream therapeutic application, would not exhibit the high tolerance to systemic LPS as murine subjects [63]. Generally, therapy along this axis alone may not represent a therapeutic strategy dictated by sustainability and plasticity for different types of tumors.

Comparison between therapeutic effects of purified LPS and bacteria was drawn from inoculi standardized according to maximal tolerated doses. Sub-lethal doses of 50 μg purified LPS and 5×10^6^ salmonellae were applied, both of which caused significant systemic TNF-α responses, 100% CT26 regression, and retardation of RenCa tumor growth. Based on stoichiometric calculations considering between 2–50 fg LPS per cell [64–66], 5 × 10^6^ salmonellae would retain between 10–250 ng bacterial derived LPS. By this estimation, *Salmonella* exerted therapeutic effects at a much lower concentration of LPS. However, in viable *Salmonella*, LPS represents only one of many immunogenic components including PAMPs which contribute to a therapeutic effect, and are constitutively supplied in concert with turnover and colonization. Signal transduction may differ depending on different immunogenic profiles, and hence also the therapeutic phenotype concerning innate cytokine and effector responses may differ between bacteria and purified LPS [39, 67, 68]. Deriving conclusions from a comparison may be further complicated by a difference in *in vivo* stability and biochemistry of different supply routes of LPS, considering as example micelle formation and aggregation of purified LPS. Nevertheless, principles demonstrated for LPS may be translated to more complex bacteria, if not directly compared in terms of stoichiometry.

At no point did TNF-α alone or as supplement exceed the therapeutic effects of bacteria alone. In accordance, neither SL7207 nor the less potent Symbioflor-2 [36] gained advantage by supplementation of TNF-α. This suggests that TNF-α induction by bacteria already retains the maximum therapeutic effect along this particular axis. While indisputably the most dominating cytokine induced by infection in this model system, a diversity of additional expressed and modulating cytokines like IL-6, IL-12, MCP-1, IFN-β, IFN-γ and IL-10 may also contribute to the therapeutic outcome with bacteria [16, 69].

The inefficiency of rTNF-α against RenCa compared to SL7207 would imply that bacterial therapeutic effects observed are elicited along a different pathway in this tumor model. Dependence on viability would also suggest that these therapeutic effects are ascribed to other than the immunogenicity of the bacterium alone. This would have been vastly preserved with the avitalization procedures. Explanations may still include local induction of TNF-α in the tumor or induction of alternate cytokines that may add therapeutic effects. More direct therapeutic effects may be ascribed to SL7207 tumor colonization or persistence in alternate organs like liver and spleen [17, 36, 37]. Altogether, this illustrates the pluripotency of SL7207. A highly plastic intrinsic ability of tumor colonization described for gram-negative bacteria like the *Salmonella* strain SL7207 may offer the needed consolidation [70]. As specific bio-vehicles delivery of recombinant immune stimulatory or toxic molecules may complement the intrinsic adjuvant effect, thus providing a more tailored and flexible therapeutic approach [1, 22, 71–73].

Considering that immune therapeutic effects of TNF-α proved exhaustible, and that such may be naturally detrimental, one should place focus on its physiological property rather than immune stimulatory capacity. Our results reinforced the role of TNF-α in tumor invasion [16, 74], and first hand demonstrated its capacity to support colonization with lower infection doses. With an altered strategy in BMTT that focuses on tumor targeting, this synergistic effect may find useful application. Particularly, as a mean to minimize adverse effects like dissemination with higher doses of *Salmonella*, while ensuring full tumor targeting capacity.

In conclusion, reduction of SL7207 to an endotoxic effect did not prove feasible in tumor models other than CT26. Nevertheless, this model highlighted that a tumor specific CTL response can be stimulated along the LPS/ TLR4 axis alone. Efficient BMTT requires more than an exhaustible adjuvant effect, thus favoring the need for complementary effects through recombinant strengthening. Focus on alternate intrinsic bacterial qualities such as tumor targeting may thus prove essential to prevail as a sustainable type of therapy.

## MATERIALS AND METHODS

### Ethics statement

All animal experiments were performed according to the guidelines of the German Law for Animal Protection and with permission by the local ethics committee and authority LAVES (Niedersächsisches Landesamt für Verbraucherschutz und Lebensmittelsicherheit). The animal permission was issued under number: 33.9-42502-04-12/0713.

### Animal models and tumor development

Six to eight weeks old female BALB/c mice were purchased from Janvier, and Rag1^−/−^of the same age and gender were made available from our in-house breeding. All mice were kept in IVC cages under SPF conditions. Upon acclimatization, mice received a s.c. injection into the right flank with 5 × 10^5^ CT26 colon carcinoma cells (ATCC CRL-2638), 1.5 × 10^6^ renal carcinoma (RenCa) cells (ATCC CRL-2947) or 5 × 10^5^ F1.A11 fibro-sarcoma cells (curtesy of Dr. Pablo Becker, HZI, Braunschweig) in 100 μl PBS (Supplementary Table 1). Upon 8 and 10 days of development for RenCa and CT26, respectively, having reached tumor sizes of 100-150 mm^3^, mice were grouped and standardized for mean and variance in tumor volume prior to injection treatments. Tumor development was expressed via volume calculations based on caliper measurements in two dimensions: V = π/6 × h × w^2^, wherein “h” = height and “w” = width.

### Inoculation procedures

*Salmonella enterica* serovar Typhimurium variant SL7207 (*hisG*^−^, Δ*aroA*) [75], was deployed from our in-house stock, and *E. coli* probiotic Symbioflor-2 (DSM 17252), a combination of strains G1/2, G3/10, G4/9, G5, G6/7 and G8 (DSM 16441-16447, resp.) [76], was provided with curtesy of Dr. Kurt Zimmermann, SymbioPharm GmbH. Inoculi were prepared as described in Kocijancic et al. (2016). In short, cultures harvested at mid log phase were washed twice, before adjusted via OD_600_ to the standard dose of 5 × 10^6^/ 100μl in pyrogen-free PBS. Preparations of avitalized bacteria were achieved via heat-inactivation at 60°C for 1 hour, or UV irradiation exposing the suspension under a laminar flow bench for 2 hours. Plating controls were performed to verify the inocula. Ultimately, bacterial suspensions were administered i.v. via the lateral tail vein at day 0 post infection (0 dpi). For alternate inoculations, LPS from *S. typhosa* and *E. coli* O55:B5 were purchased from Sigma, and administered i.v. at a dose of 50 μg, determined as a sublethal dose conversely. Recombinant murine TNF-α (Chemicon) was inoculated i.v. at an effective dose of 1 μg/ mouse [16].

### Bacterial recovery from tissues

At time points of interest, mice were sacrificed using CO_2_. Tissues of interest; tumor, spleen and liver, were aseptically isolated and suspended in 1 ml sterile PBS containing 0.1% (v/v) Triton X-100. For determination of CFU, tissues were homogenized using a gentleMAX dissociator (Miltenyi Biotec), plated in serial dilutions on selective agar, and incubated ON at 37°C.

### Measurement of TNF-α

Blood samples were acquired via heart punctuation, or retro-orbital bleeding (kinetics), around the time point 1.5 hpi. Serum was isolated using Microvette serum tubes (Sarstedt) according to manufacturer protocols. Quantification was performed using the bioassay described in Leschner et al. (2009), or the TNF-α specific ELISA Max^TM^ Standard kit (Biolegend), as specified.

### Adoptive transfer experiments

As highlighted in Figure 2A, spleens were isolated from infected, tumor bearing, or naive BALB/c mice. Tissues were disrupted using a cell strainer, and splenocytes were deprived of erythrocytes using ACK lysis buffer. Eventually CD4^+^ and CD8^+^ T cell populations were purified using negative isolation kits (Invitrogen), and verified using flow cytometry by staining for CD3, CD4, CD8, and CD19. Fractions of transferred T cells contained < 0.3% B cells, and were clear of opposing CD4^+^/ CD8^+^ T cell population (data not shown). For reconstitution experiments, 3 × 10^6^ T cells were adoptively transferred via i.v. injection into Rag1^−/−^recipients.

### Histology

Upon isolation, specimens were fixed with 4% (v/v) formalin for 24 – 48 h, and embedded in paraffin. 3 μm thin sections were stained with hematoxylin/ eosin according to standard laboratory procedures. Immune histochemical staining was performed using the following antibodies: rabbit-anti-Ki-67 (Neo Makers, RM-9106-S), rabbit-anti-pimonidazole (HP3-100 kit, Hydroxyprobe Inc.), rabbit-anti-myeloperoxidase (MPO, ThermoScientific) and DAB (3,3-Diaminobenzidine Zytomed Systems DAB530) as chromogen. Hematoxylin was used for counterstaining. Sections were analyzed by light microscopy blinded to the experimental groups.

### Statistical analyses

The non-parametric Mann-Whitney test and one-way analysis of variance (ANOVA) with Bonferroni posttests were used to compare two or more groups, respectively. Significance levels of *p* < 0.05, *p* < 0.01, or *p* < 0.001 were indicated using asterisks: *, **, and ***, respectively. All results displayed are representative of at least two independent experiments.

## SUPPLEMENTARY MATERIALS FIGURES AND TABLES


