# Supplementary material for: Therapeutic benefit of Salmonella attributed to LPS and TNF-α is exhaustible and dictated by tumor susceptibility
Source: Oncotarget. 2017 Apr 6;8(22):36492–508. doi: 10.18632/oncotarget.16906 (PMC5482671; doi:10.18632/oncotarget.16906)
Supplement: Supplementary file 1 [file oncotarget-08-36492-s001.pdf]

Therapeutic benefit of *Salmonella* attributed to LPS and TNF-α is exhaustible and dictated by tumor susceptibility

Supplementary Materials

Supplementary Table 1: Tumor cell lines used in this study

Cell line	Type	Origin and feature	Ref.
CT26	Colon carcinoma	BALB/c. Induced through N-nitro-N-methylurethan injection	ATCC CRL-2638
RenCa	Renal cortical adenocarcinoma	BALB/c. Spontaneously arisen	ATCC CRL-2947
F1.A11	Fibrosarcoma	BALB/c. Spontaneously arisen (F1). Later transduced with LBSN retroviral vector to express β-galactosidase.	[1]

[1]. Paglia P, Arioli I, Frahm N, Chakraborty T, Colombo MP, Guzman CA. The defined attenuated Listeria monocytogenes delta mp12 mutant is an effective oral vaccine carrier to trigger a long-lasting immune response against a mouse fibrosarcoma. Eur J Immunol. 1997; 27:1570–5.

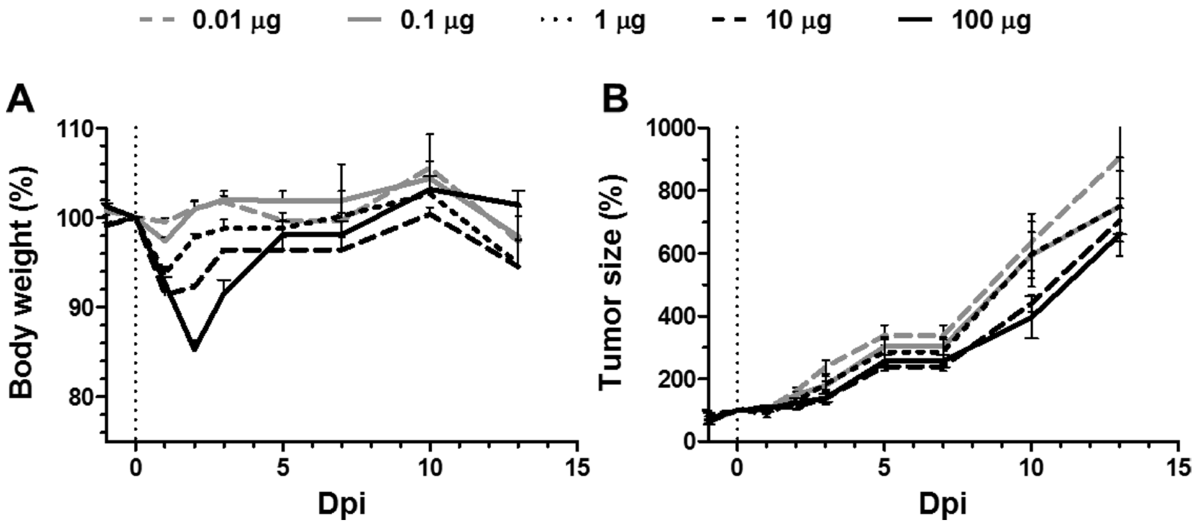

Supplementary Figure 1: Dose response to purified LPS on RenCa development and body weight. WT BALB/c mice bearing RenCa tumors were subjected to titrated doses of purified LPS. Host body weight (**A**) and tumor development (**B**) was monitored via weight- or caliper measurements in two dimensions, respectively. Tumor size is given as % volume relative to tumor volume at 0 days post infection (dpi). Therapeutic effects as well murine body weight exhibit a dose response to LPS. Displayed are Mean ± SEM. Results are representative of at least two independent experiments.

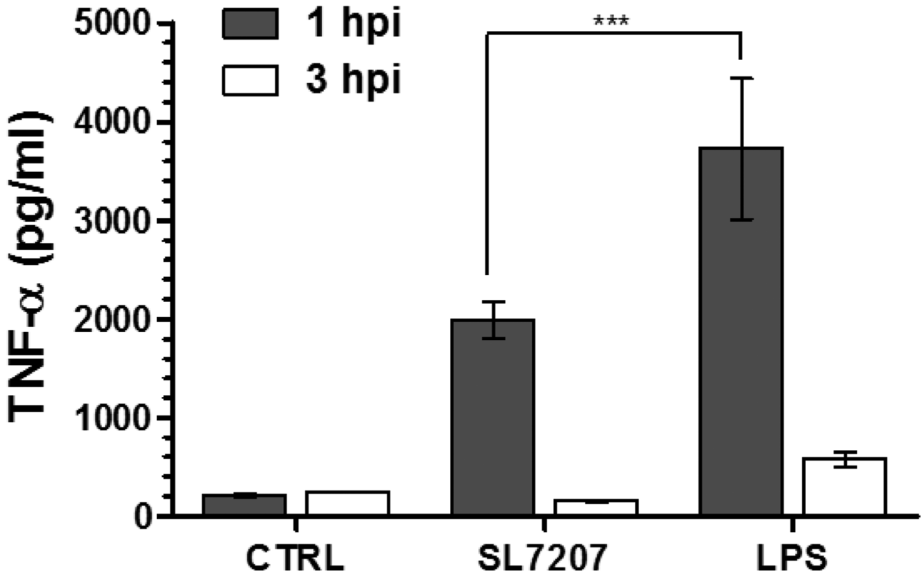

Supplementary Figure 2: Early peak of TNF-α post inoculation with *Salmonella* or LPS. BALB/c mice treated with 5e6 SL7207 or 50 µg LPS were analyzed for levels of TNF-α in sera at 1.5 and 3 hpi. Similar kinetic of TNF-α induction between bacterial infection and purified LPS. Displayed are mean ± SEM.

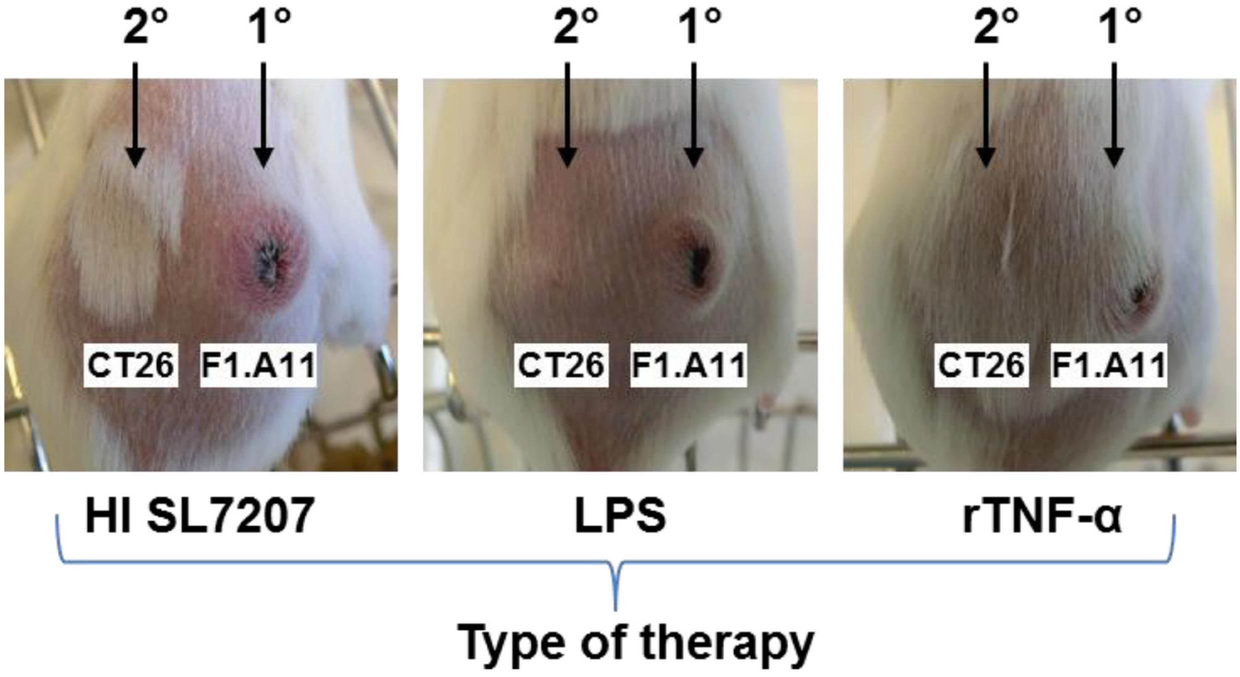

Supplementary Figure 3: Memory response against CT26 evoked upon treatment with avitalized SL7207, purified LPS or recombinant TNF-α. CT26 tumor bearing BALB/c mice which had recovered via treatments using HI SL7207, LPS or rTNF-α were rechallenged with CT26 cells. F1.A11 cells were inoculated as control. Pictures shown are representative of three individual replicates and taken on d. 12 of tumor development. All types of therapy evoke an anti-tumor memory response prohibiting secondary (2°) establishment of CT26 tumors.

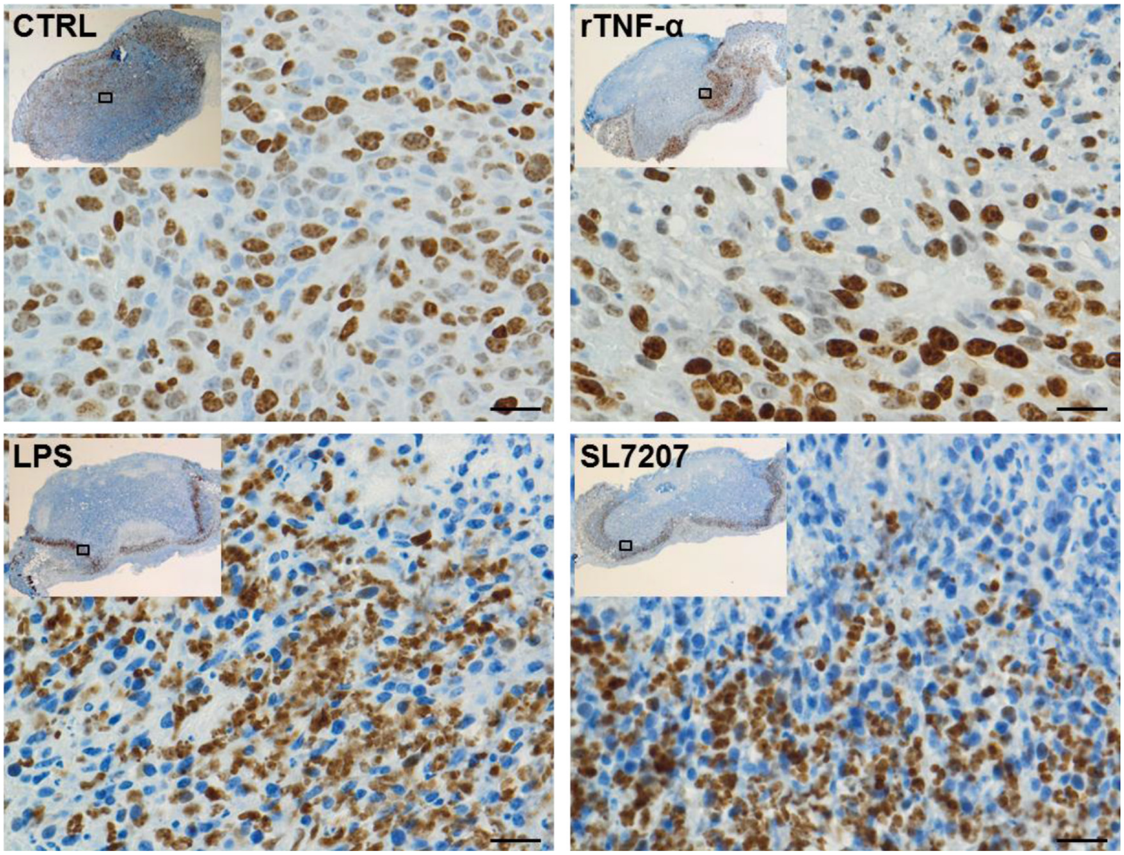

Supplementary Figure 4: Origin of proliferative activity in CT26 and RenCa immune therapy. WT BALB/c mice bearing CT26 tumors were subjected to treatments, as indicated. 48 hpi, tumors were isolated, sectioned and stained for Ki67. Morphology of the positively stained cells suggests a predominance of proliferative tumor cells. Scale bar corresponds to 20 µm. Representative images are displayed.

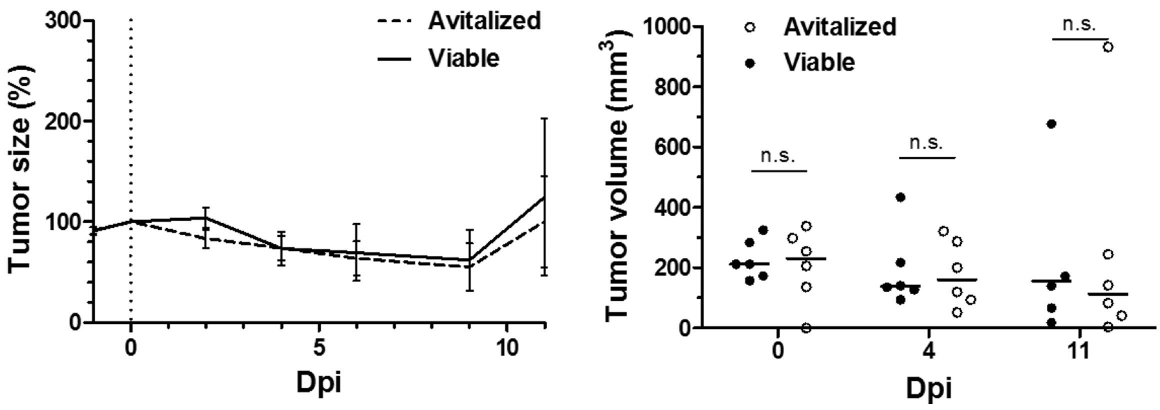

Supplementary Figure 5: Similar therapeutic efficacy against CT26 between viable and heat-inactivated probiotic *E. coli*. CT26 tumor bearing WT mice were infected with 5×10^6 viable or heat inactivated *E. coli* Symbioflor-2, before evaluating the effects on tumor growth. Non-significant difference between treatments. $N = 5$. Mean ± SEM.

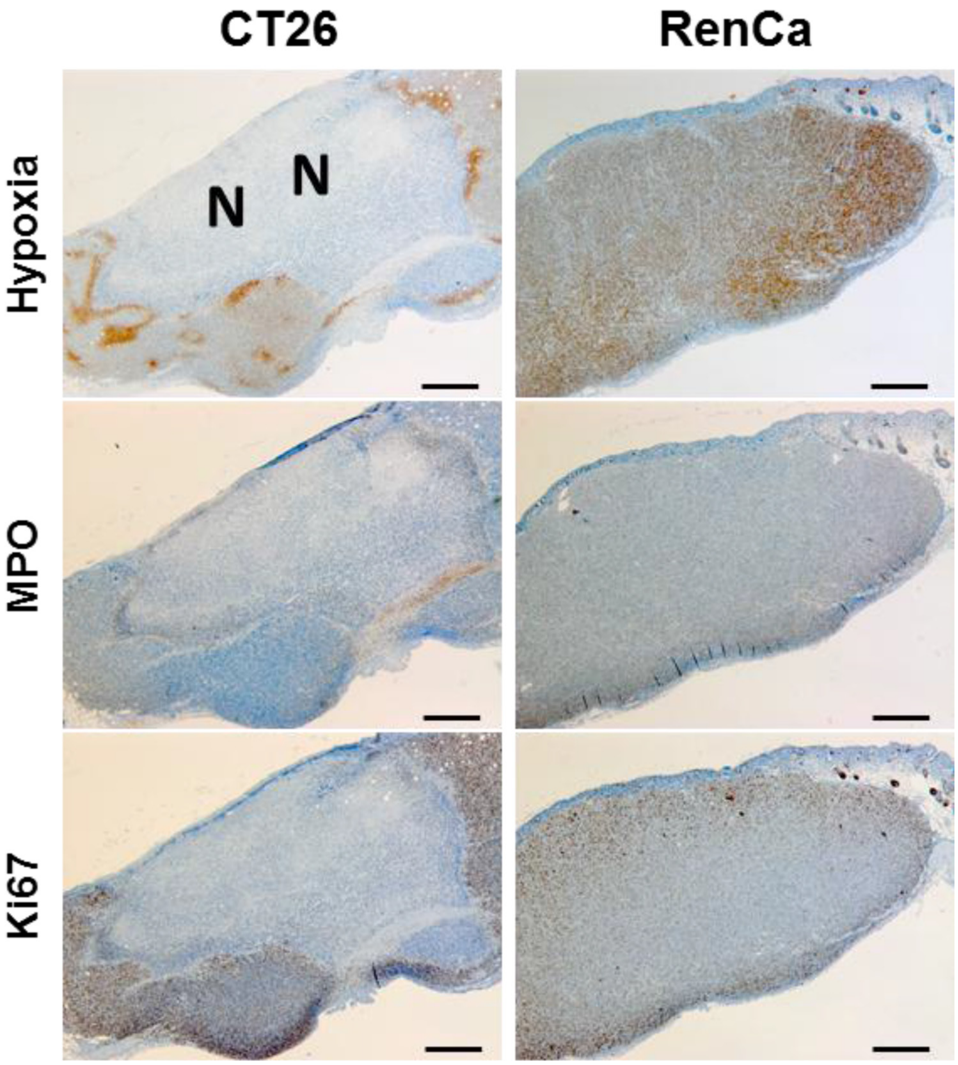

Supplementary Figure 6: Differential histological profile of CT26 and RenCa tumors by heat-inactivated SL7207. Tumor bearing BALB/c mice were subjected to treatment with avitalized SL7207, heat inactivation at 60°C for 1 hour. 48 hpi tumors were harvested, sectioned and subjected to immune-histochemical staining. “N” indicates areas of necrosis. More widespread manifestation of necrosis in the CT26 tumor compared to RenCa. Hypoxia was stained with antibodies against metabolites of pimonidazole-HCl, otherwise administered i.v. 30 mins prior to isolation. Myeloperoxidase (MPO) denotes presence of neutrophilic granulocytes, and Ki67 the extent of proliferative activity. Differential staining was performed on consecutive sections. Scale bar corresponds to 100 µm. $N = 3$. Representative images are displayed.

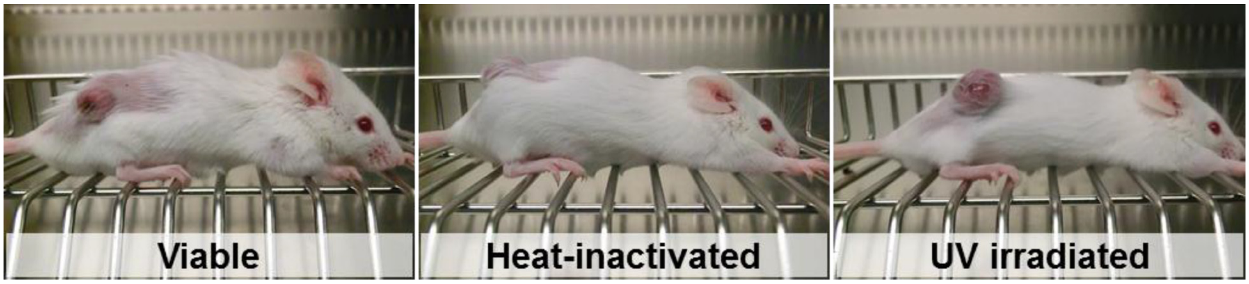

Supplementary Figure 7: Phenotypic appearance of wild-type mice treated with viable or avitalied SL7207. Photographs of tumor bearing mice having undergone treatment with 5 × 10^6 viable, heat inactivated or UV irradiated SL7207. Viable infection causes a disrupted fur coat, fatigue and disorientation whereas avitalized treatment causes no marked phenotypic change. Photos were taken 4 dpi. Representative images are displayed.

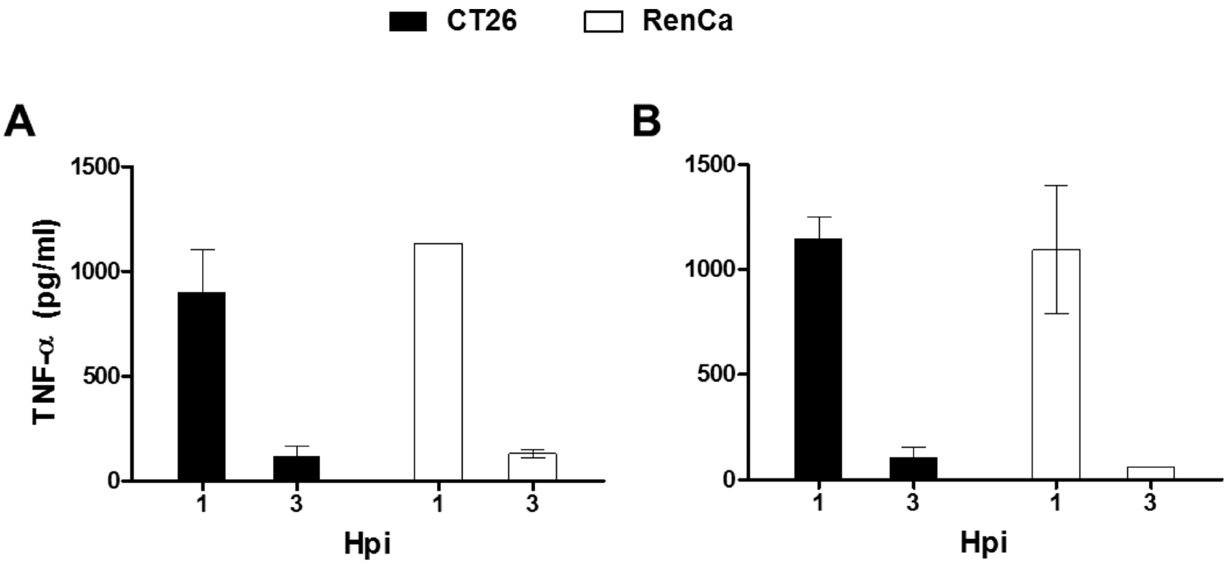

Supplementary Figure 8: Similar TNF-α response in CT26 and RenCa tumor bearing mice upon infection with probiotic *E. coli* and SL7207. Tumor bearing WT mice were infected with 5 × 10^6 SL7207 (**A**) or *E. coli* Symbioflor-2 (**B**). Sera from indicated time points were analyzed for levels of TNF-α using ELISA. Comparable kinetic and levels of TNF-α expression in CT26- and RenCa bearing mice. *N* = 3. Displayed are Mean ± SEM. Results are representative of three separate experiments.
